# Quality of life (QoL) among COVID-19 recovered healthcare workers in Bangladesh

**DOI:** 10.1186/s12913-022-07961-z

**Published:** 2022-05-30

**Authors:** Md Utba Rashid, Md Abdullah Saeed Khan, Koustuv Dalal, Soumik Kha Sagar, Mosharop Hossian, Sabrina Yesmin Barsha, Miah Md. Akiful Haque, Mohammad Ali Hossain, Mohammad Hayatun Nabi, Mohammad Delwer Hossain Hawlader

**Affiliations:** 1 Nutrition and Clinical Services Division (NCSD), International Centre for Diarrhoeal Disease Research, Bangladesh (icddr,b), Mohakhali, Dhaka, 1212 Bangladesh; 2 Department of Public Health, North South University, Bashundhara, Dhaka, 1229 Bangladesh; 3 Infectious Disease Hospital, Mohakhali, Dhaka, 1212 Bangladesh; 4 School of Health Sciences, Division of Public Health Science, Mid Sweden University, 851 70 Sundsvall, Sweden; 5 Public Health Professional Development Society (PPDS), Dhaka, 1215 Bangladesh; 6 Ibn Sina Medical College Hospital, Kollyanpur, Dhaka, 1216 Bangladesh

**Keywords:** Bangladesh, COVID-19, Quality of life (QoL), Healthcare workers, WHOQOL-BREF

## Abstract

**Background:**

The Coronavirus Disease 2019 (COVID-19) caused by the SARS-CoV-2 virus has taken the lives of more than 100,000 healthcare workers (HCWs) so far. Those who survived continuously work under immense physical and psychological pressure, and their quality of life (QoL) is impacted. The study aimed to assess the QoL among HCWs in Bangladesh who recovered from COVID-19.

**Methods:**

This cross-sectional, telephonic interview-based study was conducted among 322 randomly selected HCWs from Bangladesh who were positive for COVID-19 and recovered from the infection before the interview. Data were collected from June to November 2020. We examined the impact of COVID on the QoL of the participants using the validated Bangladesh version of the World Health Organization (WHO) Quality of life questionnaire brief (WHOQOL-BREF). All analyses were done by STATA (Version 16.1).

**Results:**

More than half of the health care professionals were male (56.0%), aged between 26–35 years (51%), and completed graduation (49%). The majority of the study participants in the four domains were married (*n* = 263, 81%) and living in Dhaka. The average score of the participants was 70.91 ± 13.07, 62.68 ± 14.99, 66.93 ± 15.14, and 63.56 ± 12.11 in physical, psychological, social relationship and environmental domains, respectively. HCWs in urban areas enjoyed 2.4 times better socially stable lives (OR: 2.42, 95% CI: 1.18–4.96) but 72% less psychologically satisfactory lives.

**Conclusion:**

HCWs' post-COVID quality of life depended on variable interaction of demographic socioeconomic, including old age, female sex, graduation, and higher monthly income. The findings indicate the issues which should be addressed to improve the quality of life of frontline workers who fight against the pandemic.

**Supplementary Information:**

The online version contains supplementary material available at 10.1186/s12913-022-07961-z.

## Background

The Coronavirus Disease 2019 (COVID-19) caused by the SARS-CoV-2 virus was first identified in China in late 2019 as a cluster of unexplained pneumonia cases. Since then, it has spread worldwide and become a global public health concern by infecting millions of people and claiming hundreds and thousands of lives [1, 2]. With high transmission capability, numbers of mutations, and other associated factors, the impact of this virus outspreads physical health and encompasses mental health, social functioning, and environmental safety [3, 4]. Since its emergence, the pandemic is constantly putting tremendous pressure on healthcare systems worldwide. Healthcare workers (HCWs) are on the frontlines to combat this invisible enemy. Nevertheless, managing an exponentially expanding number of COVID-19 patients has placed them at a high risk of exposure [5].

A Chinese study reported 3000 HCWs infected (3.8%) with five deaths by early February 2020. In Italy, this rate jagged to 10.5% in late April, and 157 HCW deaths were documented in England until early May 2020 [6–8]. Hence, the fear of infection with its consequences and unforeseen interruptions to everyday life has put the front liners in a stressful situation. In Bangladesh, where there was a scarcity of HCWs from the very beginning (according to WHO, 3.05 doctors and 1.07 nurses are available per 10,000 populations in Bangladesh) [9], battle against COVID-19 with higher patient load in grinding [10]. Although exact data could not be extracted, various sources suggest that more than 5,000 HCWs have been infected, and more than 300 have died over one year since the first case was diagnosed in Bangladesh on March 8, 2020 [11]. Moreover, COVID-19 recovered HCWs suffered significant long-term symptoms that impaired their quality of life. Studies have revealed that, notwithstanding the long-term recovery, a few COVID-19 survivors experience long-term consequences such as lung fibrosis, debilitating chronic symptoms, and psychological issues, negatively impacting their quality of living [12–14].

According to World Health Organization (WHO), "an individual's perception of their position in life in the context of the culture and value systems in which they live and concerning their goals, expectations, standards, and concerns are defined as the quality of life" [15]. Previous researchers concluded that COVID-19 might lead to decreased health-related quality of life (HRQoL) by significantly impairing life's physical and psychological domain [16–18]. However, the importance of evaluating the QoL of HCWs during their recovery period was often overlooked and underreported. Nevertheless, HCWs may have poorer clinical outcomes than non-HCWs because they are exposed to high SARS-CoV-2 density environments, work longer hours, and are more prone to psychological trauma [19]. Long term clinical consequences of COVID-19 could be linked to disruption of HCWs’ professional, psychological, social and environmental wellbeing [20, 21]. HCWs have been reported to have a high mental health impact of COVID-19, requiring professional mental health support [20, 22–24].

A significant decline in QoL was observed among people who worked in healthcare facilities during previous pandemic periods [21]. However, the number of studies to explore the QoL among HCWs recovered from COVID-19 is limited. Identifying their QoL is essential to planning their early recovery and return to hospitals. Therefore, we undertook this study to evaluate the quality of life (QoL) and their related determinants among HCWs in Bangladesh who recovered from COVID-19.

## Methods

### Study design and participants

From June 2020 to November 2020, we conducted this research on the COVID-19 positive HCWs identified and confirmed to be such by Reverse Transcription-Polymerase Chain Reaction (RT-PCR) who had either improved clinically or tested negative for the virus. For three days, clinical recovery was classified as a consecutive absence of fever, cough, or respiratory distress for mild to moderate pneumonia patients and released with hospital advice. For the asymptomatic patients, passing 14 days after the first diagnosis was regarded as a clinically cure. The study excluded those treated for COVID-19, pregnant women, and the critically ill. In our earlier nationwide study, we collected a list of COVID-19 positive cases from the whole country [25]. We randomly approached 4584 patients and then completed an interview of 3,244 participants from the list. The detailed study methods were described elsewhere [25]. Among those 3,244 participants, we found 322 HCWs and considered them recruited in this analysis. As our study response rate was 60%, we had to approach a total of 527 HCWs, of whom 134 respondents refused, 45 individuals were unreachable, and 26 participants were left before completing the questionnaire at the time of the interview. We used the Bangla validated version of WHOQOL-BREF [26] to measure the quality of life of HCWs and questionnaires related to socio-demography developed by our research team.

### Sociodemographic information, symptoms, and comorbidity profile

To assess the QoL of the HCWs, we collected participants' sociodemographic information, including age, gender, domicile, religion, educational attainment, marital status, and financial condition. In addition, we also recorded the details of their COVID-19 related hospitalization history, personal habits, presence of any chronic diseases such as heart disease, hypertension (HTN), asthma/chronic obstructive pulmonary disease (COPD), diabetes mellitus (DM), chronic kidney disease (CKD), and cancer. Symptoms that may arise or persist in the post-COVID period were also documented during the survey.

### WHOQOL-BREF

We utilized WHOQOL-BREF, a brief validated version of the WHOQOL-100 quality of life assessment questionnaire [27], to assess the QoL of COVID-19 recovered HCWs. The WHOQOL Group collaborated with fifteen foreign field centres to develop the later instrument to create a QoL evaluation that could be used across cultures. This 26 item QOL instrument has shown good to excellent psychometric properties and is cross-culturally sensitive. The WHOQOL-BREF generates a profile and score for each of the 4 QOL domains; questions are centred around the meaning respondents attribute to each aspect of life and how problematic or satisfactory they perceive them. The Physical Health domain questions are based on daily activities, medical aid, energy, mobility, the extent of pain, sleeping pattern, and working capacity. The Psychological domain focuses on participants’ personal beliefs, positive and negative feelings, self-esteem, body image, thinking, and learning capabilities. The Social Relationships domain explores the respondent’s overall satisfaction with their personal and social life. Lastly, the environment domain comprises questions about safety and security, contentment with one's property and physical surroundings, finances (does one have enough money to satisfy one's requirements), access to the necessary care, information, and transport. Moreover, the questionnaire has two specific questions regarding participants’ opinions regarding their overall QoL and health. We used the Bangla validated version of the original WHOQOL-BREF [26] questionnaire.

### Study procedure

Considering the present pandemic situation, we performed interviews over the phone. We approached all the randomly selected HCWs and recorded the response of those who felt comfortable participating in the survey. Besides, we informed the participants that there was no correct or wrong answer before the interview. Items that were misunderstood were replied, and interviewees were motivated to respond to the questions as they saw fit. The WHOQOL-BREF section of the questionnaire was scored following the manual [15]. The internal consistency of the WHOQOL-BREF questions was weighed using Cronbach's alpha. The strength of association of the questions was good among our respondents (Cronbach's alpha = 0.86).

### Statistical analysis

We applied descriptive and inferential methods to describe the quality of life of the Covid-19 recovered healthcare workers and explore the determinants of QoL among them. Statistical software STATA (Version 16.1) was used for statistical analysis. QoL scores were explored separately in four domains: physical, psychological, social, and environmental. Analyzing variance (ANOVA) models and verifying the normality assumption allowed us to compare continuous variables across different categories. We performed an independent sample t-test to compare the means of two continuous variables. We used frequencies (percent) to describe the categorical variables and chi-square tests to determine the associations between groups. For binary logistic regression analysis, QoL scores were translated into binary scores by treating a value greater or equal to 50 as 1 ("good"), otherwise 0 ("poor"). All tests were two-tailed, and *p*-values less than or equal to 0.05 were considered statistically significant.

## Results

Table 1 illustrates the sociodemographic variable of the study respondents. Among 322 health care professionals, more than half were male (*n* = 180, 56%), aged between 26–35 years (51%), and completed graduation (*n* = 158, 49%). Physicians accounted for 68% of our respondents, nurses for 27%, and others for 5% (such as laboratory technicians, pharmacists). The majority of the study participants were married (*n* = 263, 81%), urban dwellers (*n* = 247, 77%) and living in Dhaka (*n* = 154, 48%). According to the income distribution, most participants (*n* = 103, 35.64%) earned 20,000–40,000 BDT per month. Diabetes (16%), asthma/COPD (16%), and hypertension (15%) were the most prevalent comorbidities, followed by heart disease, cancer, and chronic kidney disease. One-fourth of the interviewee was smokers/past smokers (*n* = 80, 25%). Around 43% of HCW (*n* = 137, 42.55%) had to be hospitalized due to COVID-19 severity, while the other COVID-19 infected health care professionals were at home/institutional isolation during the whole infection period.

**Table 1:** Socio-demographics variable of the study participants (*n* = 322)

Name of Variable	Frequency (%)
**Age group (in years)**	
< 26	40 (12%)
26–30	96 (30%)
31–35	69 (21%)
36–40	45 (14%)
41–45	37 (12%)
46 +	35 (11%)
**Gender**	
Male	180 (56%)
Female	142 (44%)
**Division**	
Barisal	32 (10%)
Chattogram	70 (22%)
Dhaka	154 (48%)
Khulna	5 (2%)
Mymensingh	18 (6%)
Rajshahi	14 (4%)
Rangpur	12 (4%)
Sylhet	17 (5%)
**Residence**	
Rural	44 (14%)
Urban	247 (77%)
Semi-urban	31 (9%)
**Religion**	
Muslim	255 (79%)
Non-Muslim	67 (21%)
**Educational status**	
Below Graduation (SSC/HSC)	104 (32%)
Graduation	158 (49%)
Post-graduation	60 (19%)
**Marital status**	
Single	57 (18%)
Married	263 (81%)
others	2 (1%)
**Income (BDT)**	
< 20,000	56 (19%)
20,000–40,000	103 (36%)
4000–60,000	56 (19%)
60,000 +	74 (26%)
**Smoking status**	
Never smoked	242 (75%)
Current smoker	58 (18%)
Past smoker	22 (7%)
**Chronic disease**	
Hypertension	48 (15%)
Diabetes	51 (16%)
Asthma/ COPD	50 (16%)
Heart disease	23 (7%)
Chronic kidney disease	9 (3%)
Cancer	12 (4%)
**Hospital admission (Yes)**	137 (43%)

**Fig. 1:**
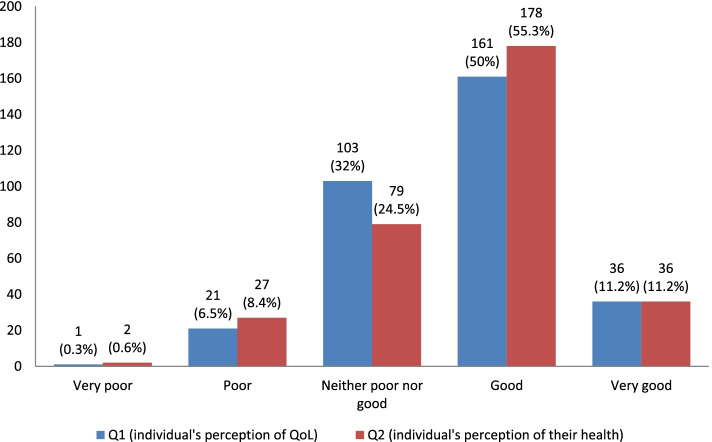
Individual’s overall perception of QoL and health of the study respondents

We found the mean score of individual's overall perception of QoL and their health (as assessed by Q1 and Q2, scored in a range of 1 to 5) were 3.65 ± 0.78 and 3.68 ± 0.81, respectively, which were slightly higher than the possible middle score (i.e., 3) (Fig. 1). The mean domain-specific scores of QoL were observed highest in the physical domain (70.91 ± 13.07), followed by social relationships (66.93 ± 15.14), environmental (63.56 ± 12.11), and psychological domain (62.68 ± 14.99) (Table 2).

**Table 2:** Overall and domain specific score averages of the survey respondents

WHOQOL-BREF		Overall mean	SD
Overall 1 (Q1)	Individual’s overall perception of QoL	3.65	0.78
Overall 2 (Q2)	Individual’s overall perception of their health	3.68	0.81
Domain 1	Physical	70.91	13.07
Domain 2	Psychological	62.68	14.99
Domain 3	Social relationships	66.93	15.14
Domain 4	Environmental	63.56	12.11

We observed a significant difference in the physical and psychological QoL scores among our HCW in different age groups (*p* = 0.001 and *p* = 0.0004, respectively) (Table 3). In both domains mean score of QoL deteriorated with the increase of age. We noticed average QoL score of Covid-19 recovered female HCWs was significantly lower than their male counterparts in psychological, social relationships, and environmental domains (*p* < 0.05). Interestingly, the mean scores of psychological, social relationships and environmental domains of HCWs differed significantly based on their corresponding division. Place of residence was a significant factor in modifying the social relationships domain (*p* < 0.05), and HCWs from urban areas scored highest in this sector. Respondents with a postgraduate level of education had a better environmental domain score than that of lower educational categories. While single HCW had a better physical and psychological QoL than married and divorced HCW, married respondents had a better social life. However, divorced/widowed HCWs lived the worst quality of life in both scenarios. As expected, study subjects hospitalized due to COVID-19 had a considerably lesser physical and psychological QoL than the home quarantined participants (*p* < 0.001). We observed proportionate deterioration of QoL scores due to chronic diseases in physical, psychological, and social relationships domains (*p* < 0.05). We also noticed the individual disease-specific worsening of QoL scores in each domain due to these chronic diseases. The analysis results were provided in the supplementary files (Supplementary 1).

**Table 3:** Comparison of individual domains score against socio-demographics variables

Variables	Physical	Psychological	Social	Environmental
**Age group**				
< 26	76.83 ± 11.72	66.88 ± 14.92	64.43 ± 15.90	60.15 ± 12.76
26 – 30	71.69 ± 13.74	65.76 ± 15.72	66.67 ± 16.91	64.10 ± 12.06
31 – 35	72.01 ± 12.72	62.84 ± 13.29	66.83 ± 14.10	62 ± 12.96
36 – 40	68.47 ± 11.07^a^	59.47 ± 13.98	66.09 ± 15.07	65 ± 12.45
41 – 45	69.35 ± 12.63	53.84 ± 14.21^abc^	68.57 ± 14.87	66.32 ± 12.32
46 +	64.6 ± 13.40^a^	62.6 ± 14.51	68.09 ± 11.65	64.29 ± 7.95
p	**0.001**	**0.0004**	0.832	0.206
**Gender**				
Male	72.03 ± 13.69	64.9 ± 15.06	69.22 ± 15.68	65.11 ± 12.86
Female	69.48 ± 12.14	59.87 ± 14.48	64.03 ± 13.95	61.60 ± 12.83
p	0.082	**0.002**	**0.002**	**0.01**
**Division**				
Barisal	71.5 ± 10.25	60.72 ± 9.01	62.5 ± 12.84	59.19 ± 11.55
Chattogram	74.11 ± 14.21	67.34 ± 14.87	67.77 ± 16.20	64.76 ± 10.17
Dhaka	70.52 ± 13.18	60.36 ± 15.77^b^	68.46 ± 15.77	64.73 ± 12.72
Khulna	65.2 ± 16.45	49.8 ± 27.24	46.2 ± 12.13^bc^	65 ± 9.57
Mymensingh	70.61 ± 10.27	68.83 ± 14.37	62.5 ± 11.56	64.11 ± 15.33
Rajshahi	68.07 ± 7.13	62.14 ± 8.02	65.64 ± 11.17	55.43 ± 6.99
Rangpur	62.58 ± 15.75	70.42 ± 11.31	62 ± .9.88	57.83 ± 9.34
Sylhet	70.29 ± 13.53	60.41 ± 13.29	73.24 ± 11.84^d^	66 ± 13.01
p	0.14	**0.003**	**0.005**	**0.023**
**Residence**				
Rural	73.09 ± 12.72	66.73 ± 11.78	62.93 ± 16.64	63.73 ± 12.93
Urban	70.64 ± 12.99	61.79 ± 15.06	68.30 ± 14.89	63.83 ± 12.16
Semi-urban	69.90 ± 14.25	64 ± 17.71	61.71 ± 13.10	61.19 ± 10.51
p	0.471	0.116	**0.012**	0.52
**Religion**				
Muslim	71.46 ± 13.27	63.75 ± 15.16	66.77 ± 15.80	63.58 ± 12.46
Non-Muslim	68.81 ± 12.15	58.63 ± 13.72	67.52 ± 12.38	63.49 ± 10.73
p	0.14	**0.013**	0.719	0.958
**Educational status**				
Below graduation (SSC/HSC)	71.00 ± 12.84	62.56 ± 15.72	65.88 ± 14.66	62.60 ± 12.45
Graduation	70.91 ± 12.46	63.35 ± 13.89	66.53 ± 15.76	62.34 ± 11.71
Post-graduation	70.73 ± 15.1	61.13 ± 16.58	69.8 ± 14.14	68.45 ± 11.49^e^
p	0.992	0.620	0.252	**0.002**
**Marital status**				
Single	75.19 ± 13.23	68.44 ± 14.35	62.96 ± 12.99	63.30 ± 12.08
Married	70.14 ± 12.79	61.77 ± 14.40^a^	68.03 ± 15.21^a^	63.63 ± 12.13
Others	50 ± 8.49^ab^	18.5 ± 17.68^ab^	34.5 ± 13.44^ab^	62.5 ± 17.68
p	**0.002**	**0.000**	**0.0006**	0.975
**Income (BDT)**				
< 20,000	71.68 ± 12.49	63 ± 14.77	63.52 ± 16.85	61.27 ± 13.80
20,000–40,000	71.22 ± 13.21	64.91 ± 13.87	65.90 ± 12.80	61.28 ± 10.07
4000–60,000	70.45 ± 12.85	63.68 ± 13.98	68.39 ± 17.95	64.54 ± 12.15
60,000 +	70.28 ± 13.4	62.74 ± 16.52	66.84 ± 15.47	64.51 ± 12.85
p	0.920	0.77	0.393	0.158
**Hospital admission**				
No	73.59 ± 13.18	66.29 ± 14.45	67.97 ± 15.09	63.48 ± 12.25
Yes	67.28 ± 12.04	57.81 ± 14.37	65.52 ± 15.15	63.68 ± 11.96
p	**0.000**	**0.000**	0.151	0.881
**Smoking status**				
No	71.99 ± 13.05	63.81 ± 14.34	67.83 ± 14.49	62.40 ± 11.09
Yes	68.09 ± 12.33	57.9 ± 15.89^a^	63.59 ± 15.88	67.86 ± 15.25^a^
Past smoker	66.41 ± 13.64	62.82 ± 17.67	65.86 ± 19.17	65 ± 11.01
p	**0.030**	**0.026**	0.151	**0.007**
**Chronic disease**				
0	73.19 ± 12.25	64.45 ± 14.86	67.21 ± 14.47	62.51 ± 11.46
1	69.62 ± 14.24	62.59 ± 13.94	69.04 ± 15.49	65.21 ± 11.98
2	63.56 ± 9.33^a^	54.31 ± 15.36^a^	65.31 ± 9.73	65.44 ± 8.78
3 +	57.75 ± 8.61^ab^	51.1 ± 13.38^ab^	57.2 ± 20.66^ab^	66.8 ± 19.15
p	**0.000**	**0.0001**	**0.018**	0.188

Scores were expressed as mean ± standard deviation (SD)

*p*-value was determined by one-way ANOVA with Posthoc analysis by Tukey

*p*-value significant at < 0.05 level in comparison to a) first category, b) second category, c) third category and d) fourth category within a variable. Significant *p*-values are marked in bold

The results of the domain-specific univariate analysis were portrayed in Table 4, where we tried to detect the individual factors responsible for modifying the quality of life scores among HCWs. Participants living in urban areas enjoyed 2.4 times better socially stable lives (OR: 2.42, 95% CI: 1.18–4.96) but 72% less psychologically healthy (OR: 0.28, 95% CI: 0.10–0.81) than those respondents living in rural areas. HCWs who completed post-graduation degrees enjoyed 3.7 times more environmentally secured lives than HCWs who failed to complete graduation (3.67, 1.33–10.14). Eventually, married HCWs led to 57% less psychologically sound quality of life (0.43, 0.18–0.99) than single HCW. An almost similar trend (0.40, 0.17–0.95) was observed for respondents who earned more than 60,000 BDT per month. On the other hand, we noticed a positive trend among the HCWs in the environmental domain, making more than 40,000 BDT. As expected, participants admitted to hospitals during the infection period were 65% (0.35, 0.21–0.60) less likely to stay psychologically healthy than those who were not. Likewise, smokers were more at risk in terms of psychological (0.30, 0.16–0.56) and social (0.45, 0.24–0.87) quality of life than non-smokers. Notwithstanding, a significant deterioration of the patients' QoL was observed with comorbidities except in the environment domain.

**Table 4:** Factors associated with each domain of WHOQOL-BREF among the study participants in univariate logistic regression analysis

Variables	Physical		Psychological		Social relationships		Environmental	
	**OR**	**95% CI**	**OR**	**95% CI**	**OR**	**95% CI**	**OR**	**95% CI**
**Age group**								
< 26	1.00		1.00		1.00		1.00	
26 – 30	0.25	0.03 – 2.02	0.88	0.32 – 2.44	1.29	0.57 – 2.91	2.14	0.96 – 4.78
31 – 35	0.42	0.04 – 3.86	0.64	0.22 -1.80	**2.86**	**1.08 – 7.57**	1.37	0.60 – 3.11
36 – 40	0.36	0.03 – 3.60	0.35	0.12 – 1.03	1.98	0.71 – 5.50	**3.26**	**1.16 – 9.12**
41 – 45	0.29	0.03 – 2.93	**0.21**	**0.07 – 0.61**	2.74	0.86 – 8.75	**3.84**	**1.23 – 12.00**
46 +	**0.07**	**0.01 – 0.62**	1.06	0.29 – 3.82	2.07	0.68 – 6.28	**6.4**	**1.67 – 24.58**
**Gender**								
Male	1.00		1.00		1.00		1.00	
Female	0.83	0.39 – 1.78	0.64	0.38 – 1.07	0.64	0.37 – 1.10	0.79	0.47 – 1.34
**Residence**								
Rural	1.00		1.00		1.00		1.00	
Urban	0.51	0.12 – 2.27	**0.28**	**0.10 – 0.81**	**2.42**	**1.18 – 4.96**	1.53	0.75 – 3.14
Semi-urban	0.20	0.04 – 1.06	0.52	0.13 – 2.12	0.98	0.37 – 2.62	1.75	0.58 – 5.26
**Religion**								
Muslim	1.00		1.00		1.00		1.00	
Non-Muslim	1.29	0.47 – 3.51	0.57	0.32 – 1.05	2.08	0.94 – 4.60	1.25	0.64 -2.45
**Educational status**								
Below graduation (SSC/HSC)	1.00		1.00		1.00		1.00	
Graduation	1.57	0.63 – 3.93	1.42	0.78 – 2.58	1.08	0.59 – 1.96	0.95	0.54 – 1.68
Post-graduation	0.60	0.23 – 1.58	0.78	0.38 – 1.58	1.85	0.77 – 4.43	**3.67**	**1.33 – 10.14**
**Marital status**								
Single	1.00		1.00		1.00		1.00	
Married	0.75	0.25 – 2.26	**0.43**	**0.18 – 0.99**	1.29	0.65 – 2.58	1.18	0.60 – 2.3
Others	0.08	0.003 – 1.45	1	0	1	0	0.33	0.02 – 5.55
**Income (BDT)**								
< 20,000	1.00		1.00		1.00		1.00	
20,000–40,000	0.43	0.12 – 1.59	1.04	0.43 -2.54	1.56	0.74 – 3.31	1.87	0.93 – 3.78
40,000–60,000	0.58	0.13 – 2.54	1	0.36 – 2.74	1.64	0.68 – 3.94	**2.45**	**1.05 – 5.76**
60,000 +	0.64	0.15 – 2.69	**0.40**	**0.17 – 0.95**	1.45	0.65 – 3.23	**2.82**	**1.26 – 6.31**
**Hospital admission**								
No	1.00		1.00		1.00		1.00	
Yes	0.49	0.23 – 1.06	**0.35**	**0.21 – 0.60**	0.63	0.37 – 1.1	1.31	0.76 – 2.24
**Smoking status**								
No	1.00		1.00		1.00		1.00	
Yes	1.35	0.45 – 4.08	**0.30**	**0.16 – 0.56**	**0.45**	**0.24 – 0.87**	1.32	0.64 – 2.71
Past smoker	0.63	0.17 – 2.31	1.03	0.33 – 3.18	0.69	0.24 – 1.99	1.39	0.45 – 4.26
**Chronic disease**								
0	1.00		1.00		1.00		1.00	
1	**0.36**	**0.15 – 0.85**	0.83	0.43 – 1.59	1.18	0.58 – 2.40	1.71	0.86 – 3.42
2	0.42	0.08 – 2.08	**0.28**	**0.10 – 0.81**	1.55	0.34 – 7.09	2.25	0.50 – 10.21
3 +	**0.24**	**0.07 – 0.84**	**0.12**	**0.04 – 0.32**	**0.15**	**0.06 – 0.39**	0.60	0.23 – 1.57

OR (95% CI) shown in bold are statistically significant at *p*<0.05 level

We performed multivariable logistic regression analysis to analyze each variable against each domain to classify the critical factors related to QoL (Table 5). After adjusting the variables, we found that the participants over 46 years of age enjoyed better psychologically sound (AOR: 23.71, 95% CI: 1.31–430.32) and environmentally secure (AOR: 17.23, 95% CI: 1.88–165.52) life than the respondents aged below 26 years. Psychologically and socially, female HCWs were 74% and 59% less likely (0.24, 0.08–0.67; 0.41, 0.19–0.89) to have a good QoL than male HCWs, respectively. In urban areas, the HCW's chance of living a good-quality social life was 3.37 times higher than the rural participants (3.37, 1.37–8.30), while HCWs living in the semi-urban areas led physically impoverished life (0.07, 0.006–0.86). Besides, graduated HCWs had a 2.46 times greater probability of having a decent psychological (2.46, 1.06–5.68) QoL than those who failed to complete graduation. The participants who were married enjoyed better physical life than those single. The HCWs who earned more than 60,000 BDT monthly enjoyed better-secured lives than the participants who earned less than 20,000 BDT. Hospitalized HCWs were 86% less probability of enjoying a better QoL in the physical domain (0.14, 0.04–0.55) than those who were not. Environmental QoL was more favourable for ex-smokers than non-smokers (15.72, 1.28–192.46). Current smokers had a 76% lower chance (0.24, 0.07–0.85) of maintaining a good psychological QoL than never smoker participants. HCWs with three or more comorbidities had a worse QoL on psychological, social, and environmental measures than those without comorbidity. Finally, for each week since they recovered, people with COVID-19 were more likely to get a better physical quality of life (1.16, 1.035–1.309).

**Table 5:** Factors associated with each domain of WHOQOL-BREF among the study participants in multivariable logistic regression analysis

Variables	Physical		Psychological		Social		Environmental	
	**AOR**	**95% CI**	**AOR**	**95% CI**	**AOR**	**95% CI**	**AOR**	**95% CI**
**Age group**								
< 26	1.00		1.00		1.00		1.00	
26 – 30	0.07	0.005 – 1.06	0.93	0.25 – 3.49	0.90	0.32 – 2.55	1.39	0.51 – 3.91
31 – 35	0.07	0.004 – 1.23	0.81	0.18 – 3.64	1.25	0.36 – 4.26	0.87	0.29 – 2.63
36 – 40	0.12	0.005 – 2.84	0.43	0.08 – 2.17	1.49	0.37 – 5.93	1.52	0.38 – 6.10
41 – 45	0.10	0.003 – 3.73	0.30	0.05 – 1.60	1.72	0.37 – 7.92	2.00	0.46 – 8.77
46 +	0.05	0.002 – 1.10	**23.71**	**1.31 – 430.32**	3.99	0.64 – 25.07	**17.23**	**1.88 – 165.52**
**Gender**								
Male	1.00		1.00		1.00		1.00	
Female	1.15	0.34 – 3.87	**0.24**	**0.08 – 0.67**	**0.41**	**0.19 – 0.89**	1.04	0.52 – 2.08
**Residence**								
Rural	1.00		1.00		1.00		1.00	
Urban	0.82	0.08 – 8.28	0.26	0.06 – 1.15	**3.37**	**1.37 – 8.30**	2.22	0.90 – 5.49
Semi-urban	**0.07**	**0.006 – 0.86**	0.25	0.04 – 1.64	1.05	0.32 – 3.43	2.54	0.69 – 9.38
**Religion**								
Muslim	1.00		1.00		1.00		1.00	
Non-Muslim	3.19	0.47 – 21.55	1.44	0.52 – 4.01	1.52	0.56 – 4.12	0.72	0.30 – 1.72
**Educational status**								
Below graduation (SSC/HSC)	1.00		1.00		1.00		1.00	
Graduation	3.24	0.78 – 13.45	**2.46**	**1.06 – 5.68**	1.21	0.58 – 2.51	0.74	0.36 – 1.49
Post-graduation	0.86	0.14 – 5.20	2.76	0.67 – 11.40	1.29	0.35 – 4.77	2.77	0.63 – 12.16
**Marital status**								
Single	1.00		1.00		1.00		1.00	
Married	**7.90**	**1.35 – 46.09**	1.40	0.43 – 4.57	1.13	0.43 – 2.97	0.994	0.41 – 2.38
Others	0.28	0.004 – 20.42	1	0	1	0	0.06	0.001 – 1.94
**Income (BDT)**								
< 20,000	1.00		1.00		1.00		1.00	
20,000–40,000	0.47	0.08 – 2.86	1.26	0.38 – 4.16	1.38	0.55 – 3.43	1.70	0.72 – 3.98
4000–60,000	1.22	0.13 – 11.01	1.06	0.27 – 4.13	1.25	0.42 – 3.72	2.18	0.77 – 6.17
60,000 +	0.46	0.06 – 3.31	0.42	0.13 – 1.37	1.90	0.65 – 5.57	**2.94**	**1.09 – 7.89**
**Hospital admission**								
No	1.00		1.00		1.00		1.00	
Yes	**0.14**	**0.04 – 0.55**	0.46	0.20 – 1.03	0.66	0.32 – 1.37	1.39	0.68 – 2.86
**Smoking status**								
No	1.00		1.00		1.00		1.00	
Yes	3.90	0.57 – 26.86	**0.24**	**0.07 – 0.85**	0.53	0.18 – 1.55	3.06	0.90 – 10.38
Past smoker	6.06	0.16 – 225.92	1	0	1.06	0.22 – 5.04	**15.72**	**1.28 – 192.46**
**Chronic disease**								
0	1.00		1.00		1.00		1.00	
1	0.39	0.09 – 1.63	1.32	0.45 – 3.84	0.83	0.33 – 2.10	0.88	0.35 – 2.25
2	1.14	0.06 – 20.58	0.40	0.05 – 3.14	0.59	0.09 – 3.81	0.25	0.36 – 1.72
3 +	0.29	0.03 – 3.06	**0.08**	**0.01 – 0.55**	**0.05**	**0.01 – 0.23**	**0.06**	**0.01 – 0.32**
**Duration**^a^** (Weeks)**	**1.16**	**1.035—1.309**	1.05	0.962—1.149	1.05	0.981—1.129	1.04	0.970—1.115

*p < 0.05

^a^the time (in weeks) passed between confirmation of COVID-19 and the date of interview

AOR (95% CI) shown in bold are statistically significant at *p*<0.05 level

## Discussion

The extraordinary devastation induced by the COVID-19 pandemic has put millions of lives in danger and caused significant disruption to the financial system. Those who become infected with COVID-19 had to go through the most agonizing experiences. HCWs are the most vulnerable groups which render their service in front of the highest possible threat. So, assessing the quality of life of the COVID-19 recovered HCWs was a time-demanding necessity in the current situation.

We observed an improvement in the individuals' overall perception of QoL and their health (Q1 3.65 ± 0.78, Q2 3.68 ± 0.81) after getting recovered from the COVID-19. This positive observation was in harmony with four domains of QoL, where the physical domain scored the highest, shadowed by social relationship, environmental, and psychological domains, respectively. Eventually, the promising scores corresponding to each domain assert the overall improvement of the QoL among the HCWs.

Despite the recovered HCWs presenting with improved QoL, a variation in their domain-specific scores representing QoL was observed. To be specific, they showed significant variation concerning different sociodemographic variables, including age, gender, urban residence, higher educational attainment, marital status, higher income, past smoking and the presence of chronic disease. The findings of the earlier studies performed on the general population are congruent to the current study [16, 18, 28].

We noticed that the chance of having good psychological and environmental scores increased with increasing age when adjusted for other factors. The possible explanation for such findings is that HCWs start their careers later than other professions. Due to the lengthy education system, many HCWs do not begin earning substantial incomes until they are 45 or older, well after most of their peers from other professions [29]. Besides, with the increasing age, they find a balance between their profession and family life which he/she was initially thriving to adjust [30]. According to the medical economics report, the late establishment of the HCWs enabled them to acquire financial stability, freedom, physical safety and security, better accessibility to the wellbeing and social care, a good home environment as well as physical environment, and safe transportation in their later life [29]. As a result, despite their multimorbidity, the economic solvency of these older adults gives them a positive feeling by ruling out their negative esteem.

Our current study found that female HCWs are more vulnerable to observing a substandard psychological and social life than their male counterparts. Females experienced 31% deterioration in their psychological and social quality of life due to gender issues, in line with the previous studies conducted on normal adults of Bangladesh [31]. Furthermore, a study on Chinese HCWs [32] also discovered similar results. This might be because most of our female participants were frontline physicians or nurses who might have contracted SARS-CoV2 while treating patients and were still working for long diligent hours under high risk after recovery [33, 34]. Besides, due to the sudden COVID-19 outbreak, the government engaged junior physicians and nurses with fewer years of work experience, making their duties stressful and psychologically challenging [35–39]. During the SARS pandemic, research steered among HCWs in emergency rooms found that female HCWs were more prone than male HCWs to experience anxiety and behavioural disengagement [38].

Participants living in the urban areas had good social relationships domain scores than the rural participants. According to Shucksmith et al., rural communities led to a substantially poorer quality of life than metropolitan areas [40]. As in other countries [41], availability of modern amenities and treatment, good carrier growth, immediate social support from the surroundings, and a favourable environment might have been determinants of improved social QoL among the COVID-19 recovered urban HCWs [42]. We also found that COVID-19 recovered HCWs, residing in the semi-urban areas were significantly more prone to lead a substandard physical QoL than rural respondents. As our current study was carried out during the initial period of the pandemic, more cases were reported from the urban and semi-urban areas than the rural areas [43]. Our COVID-19 frontlines of semi-urban regions occupied inpatient management with low resources during the COVID-19 pandemic, where the patient surge was tremendous. The earlier study showed that uninterrupted workload could induce burnout among HCWs and diminish QoL [4]. In order to keep up with the ongoing surges in inpatient load, HCWs serving in COVID-19-designated units or hospitals had to deal with a dearth of skilled medical personnel and personal protective equipment, as well as inadequate training in the proper maintenance of PPE [44]. Therefore, these unexpected increases in load without adequate compensation and subsequent COVID-19 infection led to a decreased quality of life among the HCWs of semi-urban areas. The reciprocal relationships between psychological burnout, lack of resting time, and poor quality of life have been reported in HCWs managing the COVID-19 patients [8].

We found that married HCWs who recovered from COVID-19 enjoyed a better physical life than the single respondents. The presence of a person to look after during the COVID affected days might be the possible reason for their quick physical ailment in this particular domain.

A positive relationship was observed in the psychological domain between the QoL scores and the level of education among the study participants. Our findings were in line with the worldwide study conducted by Skevington et al. [45] and the study by Regidor et al. [46] performed on the Spanish population. They both reported that higher educational attainment was the key to acquiring better occupational prospects, unlocking financial stability, positive feelings, opportunities for upgrading skills, and overall a good quality of life. In line with this, we also found that graduate HCWs led 2.5 times better psychological QoL than the least educated group. However, it is plausible that graduate HCWs had more positive feelings of happiness and contentment due to their higher income, which secured a more positive environmental QoL. We can support this assumption because we noticed a positive association between the participants’ financial solvency and their environmental domain of QoL. Higher-income ensured access to better physical safety and security, excellent physical and social care accessibility, finer transportation, pollution, and a noise-free healthier environment. However, those with little education reported the worst health and financial resources and the worst overall quality of life [45].

HCWs admitted into the hospital due to COVID-19 had lower scores in the physical domain of the QoL index after recovery. The persistence of post-COVID-19 symptoms, functional disabilities, slow healing, and posttraumatic mental distress after severe infection might be the reason behind the declining physical quality of life. The persistence of symptoms after recovery was related to the patients’ low physical QoL [47], and impacted mental function [48]. Other studies found that the adverse impact on HCWs was marked compared to others [12].

As expected, we noticed that the active smokers participants were less likely to have a good score in the psychological domain of QoL. However, smoking did not affect catching the coronavirus [49] but was responsible for decreasing their self-esteem by creating negative feelings within themselves. Several studies also reported the negative effect of smoking on deteriorating QoL among the study participants [50–55]. We found that smoking quitters enjoyed a healthier environmental QoL than never smokers. The observed elevated QoL among the ex-smokers might be attributed to their self-determination, impetus from the behavioural change and improved sense of wellbeing. However, there is still the possibility of the unmeasured confounders influencing our analysis.

We observed that almost all of the six non-communicable comorbid diseases, namely HTN, DM, IHD, BA/COPD, CKD, and cancer, were responsible for significantly lower QoL scores in the physical health, psychological, and social relationship, and environmental domains of life among the HCWs. We found that the higher the number of comorbidities, the lower the chance of enjoying a satisfactory quality of life. Previous research conducted on COVID and non-COVID patients corroborates these findings [18, 49, 56–64]. As chronic diseases can exacerbate disease severity, provide a dire prognosis, and increase fatality in COVID-19 patients, the cumulative impact of COVID plus chronic disease might have led to our subjects' lower QoL even after recovery.

Lastly, the physical QoL of the COVID-19 recovered HCWs was found to be improved over time. As the physical domain solely depended on patients' physical wellbeing, the finding was relevant. On the other hand, it was evident from other research that COVID-19 exerted a tremendous negative impact on people's psychological domain [65]. Therefore, the time required to overcome psychological havoc could be a possible area to be explored in outlining a proper management plan for the patients’ early recovery.

The relatively limited number of individuals who gave data can be viewed as a drawback in the generalizability of the results. Future studies should reveal more generalizable findings by gathering data from a larger sample of HCWs. Given that the current study is a cross-sectional research case, longitudinal studies examining the pandemic's long-term impacts are warranted. The gender of the participant was found to have a substantial effect on the social and psychological QoL of the HCWs. Thus, additional gender-based comparative research examining the factors of HCWs' working conditions during the COVID-19 epidemic may better understand the issue.

## Conclusions

HCWs' post-COVID QoL was affected by various demographic and socioeconomic determinants, including their age, gender, education, and monthly salary. One or more areas of QoL were significantly impacted when disease severity and the degree of comorbidities were considered. However, all the domains of QoL improved over the period where the physical domain had been found significant. Researchers in national and worldwide communities would surely be interested in our findings, which would lead policymakers in developing particular recuperation and rehabilitation plans, initiatives, and strategies for COVID-19-affected health care workers.

## Supplementary Information

**Additional file 1:** **Supplementary 1.** Comparison of individual domain score by chronic disease status.

## Data Availability

Data could be available from Dr Mohammad Delwer Hossain Hawlader (mohammad.hawlader@northsouth.edu)

## Abbreviations

ANOVA: Analyzing variance
AOR: Adjusted odds ratio
COVID-19: Coronavirus disease 2019
COPD: Chronic obstructive pulmonary disease
CKD: Chronic kidney disease
DM: Diabetes mellitus
HCWs: Healthcare workers
HRQoL: Health-related quality of life
HSC: Higher secondary school certificate
HTN: Hypertension
OR: Odds ratio
PPE: Personal protective equipment
QoL: Quality of life
RT-PCR: Reverse Transcription-Polymerase Chain Reaction
SSC: Secondary School Certificate
WHO: World health organization
WHOQOL-BREF: World Health Organization Quality of life questionnaire brief
