# Supplementary material for: Quality of life (QoL) among COVID-19 recovered healthcare workers in Bangladesh
Source: BMC Health Serv Res. 2022 May 30;22:716. doi: 10.1186/s12913-022-07961-z (PMC9150765; doi:10.1186/s12913-022-07961-z)
Supplement: Supplementary file 1 — Additional file 1: Supplementary 1. Comparison of individual domain score by chronic disease status. [file 12913_2022_7961_MOESM1_ESM.docx]

**Supplementary 1: Comparison of individual domain score by chronic disease status**

| **Variables** | **Physical** | **Psychological** | **Social** | **Environmental** |
| --- | --- | --- | --- | --- |
| **Hypertension** | | | | |
| No | 71.71±13.04 | 63.48±14.96 | 67.08±14.91 | 63.33±11.90 |
| Yes | 66.25±12.37 | 58.10±14.52 | 66.02±16.53 | 64.88±13.32 |
| p | **0.007** | **0.022** | 0.653 | 0.416 |
| **Diabetes** | | | | |
| No | 72.45±12.63 | 64.11±14.58 | 67.62±14.81 | 62.76±11.44 |
| Yes | 62.71±12.41 | 55.08±14.99 | 63.25±16.46 | 67.82±14.60 |
| p | **0.000** | **0.0001** | 0.059 | **0.006** |
| **Heart disease** | | | | |
| No | 71.70±12.95 | 63.20±14.85 | 67.65±14.63 | 63.29±11.50 |
| Yes | 60.57±10.07 | 55.91±15.53 | 57.61±18.64 | 67.09±18.24 |
| p | **0.0001** | **0.024** | **0.002** | 0.148 |
| **Asthma/ COPD** | | | | |
| No | 72.23±12.61 | 64.03±14.66 | 67.63±14.24 | 63.34±11.49 |
| Yes | 63.7±13.27 | 55.32±14.80 | 63.1±19.05 | 64.76±15.12 |
| p | **0.000** | **0.0001** | **0.052** | 0.448 |
| **CKD** | | | | |
| No | 71.21±12.98 | 63.04±14.86 | 67.26±15.04 | 63.35±11.87 |
| Yes | 60.22±12.42 | 50.11±14.96 | 55.56±15.16 | 71±18.06 |
| p | **0.013** | **0.011** | **0.022** | 0.062 |
| **Cancer** | | | | |
| No | 71.37±13.01 | 63.29±14.86 | 67.48±14.7 | 63.17±11.62 |
| Yes | 58.83±7.84 | 47±9.06 | 62.58±19.65 | 73.67±19.28 |
| p | **0.001** | **0.0002** | **0.0008** | **0.003** |

- p-values were determined using independent sample t test
